# Novel Low-Twist Bast Fibre Yarns from Flax Tow for High-Performance Composite Applications

**DOI:** 10.3390/ma14010105

**Published:** 2020-12-29

**Authors:** Nina Graupner, Karl-Heinz Lehmann, David E. Weber, Hans-Willi Hilgers, Erik G. Bell, Isabel Walenta, Luisa Berger, Torsten Brückner, Kay Kölzig, Herbert Randerath, Albert Bruns, Bernd Frank, Maik Wonneberger, Marc Joulian, Lisa Bruns, Friedrich von Dungern, Alexander Janßen, Thomas Gries, Stefan Kunst, Jörg Müssig

**Affiliations:** 1 The Biological Materials Group, Department of Biomimetics, HSB–City University of Applied Sciences, Neustadtswall 30, D-28199 Bremen, Germany; david.weber@hs-bremen.de; 2 Institut für Textiltechnik of RWTH Aachen University, Otto-Blumenthal Straße 1, D-52074 Aachen, Germany; Karl-Heinz.Lehmann@ita.rwth-aachen.de (K.-H.L.); ErikGordon.Bell@ita.rwth-aachen.de (E.G.B.); herbert.randerath@ita.rwth-aachen.de (H.R.); alexander.janssen@ita.rwth-aachen.de (A.J.); thomas.gries@ita.rwth-aachen.de (T.G.); 3 Wenzel & Hoos GmbH, Berliner Straße 35, D-36304 Alsfeld-Leusel, Germany; hilgers@technotex.eu (H.-W.H.); stefan.kunst@wenzel-hoos.de (S.K.); 4 INVENT GmbH, Christian-Pommer-Straße 47, D-38112 Braunschweig, Germany; Isabel.Walenta@invent-gmbh.de (I.W.); Maik.Wonneberger@invent-gmbh.de (M.W.); marc.joulian@invent-gmbh.de (M.J.); friedrich.vondungern@invent-gmbh.de (F.v.D.); 5 NOVACOM Verstärkte Kunststoffe GmbH, Werkstraße 26, D-52076 Aachen, Germany; LB@novacom-ac.de (L.B.); ab@novacom-ac.de (A.B.); L.bruns@novacom-ac.de (L.B.); 6 SachsenLeinen GmbH, August-Bebel-Straße 2, D-04416 Markkleeberg, Germany; t.brueckner@sachsenleinen.de (T.B.); kay.koelzig@sachsenleinen.de (K.K.); 7 BAFA neu GmbH, Stephanstraße 2, D-76316 Malsch, Germany; Bernd.Frank@bafa-gmbh.de

**Keywords:** flax, staple fibre yarn, leaf spring, mechanical characteristics, high-performance composites

## Abstract

The use of natural fibres for components subjected to higher mechanical requirements tends to be limited by the high price of high-quality semi-finished products. Therefore, the present study deals with the development of more cost-effective staple fibre yarns made from flax tow. In the subsequent processing stage, the yarns were processed into quasi-unidirectional (UD) fabrics. The results of the fibre characterisation along the process chain have shown that no significant mechanical fibre damage occurs after slivers’ production. Fibres prepared from yarns and fabrics show comparable characteristics. The yarns were processed to composites by pultrusion to verify the reinforcement effect. The mechanical properties were comparable to those of composites made from a high-quality UD flax roving. The fabrics were industrially processed into composite laminates using a vacuum infusion and an autoclave injection process (vacuum injection method in an autoclave). While impact strength compared to a reference laminate based on the UD flax roving was achieved, tensile and flexural properties were not reached. An analysis showed that the staple fibre yarns in the fabric show an undulation, leading to a reorientation of the fibres and lower characteristic values, which show 86–92% of the laminate made from the flax roving. Hybrid laminates with outer glass and inner flax layers were manufactured for the intended development of a leaf spring for the bogie of a narrow-gauge railroad as a demonstrator. The hybrid composites display excellent mechanical properties and showed clear advantages over a pure glass fibre-reinforced composite in lightweight construction potential, particularly flexural stiffness.

## 1. Introduction

The research and development of natural fibre-reinforced composites continue to progress. In the past used as a cheap filler, today, more and more natural fibres, especially bast fibres, are being used for high-quality products and products subjected to higher mechanical requirements. Over the past years, natural fibre-reinforced composites and their structure-property relationships have been increasingly investigated, and processing techniques have continuously been optimised. Flax fibres were a particular focus of research in the context mentioned above. Many studies have now been conducted on the influence of fibre properties on composite characteristics [1,2,3,4]. Ramesh [2] reviewed plant growth, harvesting, fibre structure, properties, the effect of chemical treatments, and the influence of various factors, such as fibre length, diameter, fibre location and fibre orientation on the composite characteristics. Moreover, processing–structure–property relationships, the effect of fibre configuration, manufacturing processes, fibre load, matrix selection, ecological impacts, mechanical properties, life cycle assessment, failure studies and fibre/matrix interface parameters on the characteristics of flax fibre-reinforced composites have been analysed.

Individual studies deal, for example, with the influence of scattering fibre properties [5], yarn type [6,7,8], yarn twist [5,9,10] different types of fabrics and semi-finished products [10,11], process parameters [12], the influence of voids [13], moisture [14] or the fibre volume fraction [15] on the mechanical composite characteristics. The composites may be based on both thermoplastic and thermoset matrices, which can meanwhile also be fully (thermoplastics) or partially (thermosets) biobased [3]. In some areas, such as sports or design, high-performance composite materials made from flax are available. Unfortunately, usually high-quality long scutched flax [16] is required for these high-performance applications, which may be very expensive compared to natural fibres used; for example, for interior components in the automotive industry. The prices for biaxial flax fabrics are currently about twice as high as comparable products made of glass fibres and are nearly as high as the prices for carbon fibre fabrics [17]. Scutched flax is appreciated for its length, fineness and cleanness, while tows are entangled, impure and cheaper. Martin et al. [16] found the potential to convert flax tows from a cheap by-product to reinforcement for high-performance composites. The mechanical properties of the composites comparable to those of scutched flax fibre-reinforced composites were reported. The prerequisite for this is a suitable cleaning and aligning process of the fibres [16].

The findings mentioned before give reason to be optimistic for developing high-quality yarn from flax tow for a more cost-effective reinforcement. Therefore, this study aims to use flax tow for the production of novel staple fibre yarns. The use of hemp is also to be implemented in future work, as hemp offers even more advantages in terms of price. However, the processability of hemp into yarns, especially staple fibre yarns usable for high-performance composite applications, is much more difficult [18], for this reason, the first approach is to implement the process based on flax.

**Figure 1:**
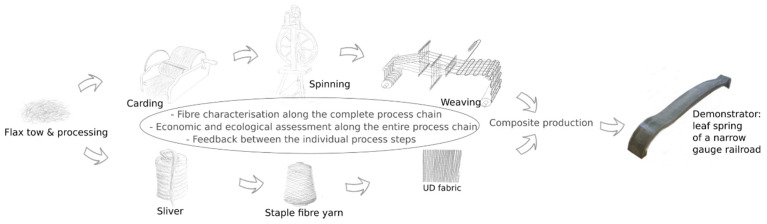
Process chain—from raw fibre to composite.

The work aims to achieve a comparable reinforcing effect in the composite with commercially available high-quality flax yarns with a low twist or unidirectional (UD) flax rovings. Composites reinforced with yarns usually perform better than composites made from fleeces or needle felts. However, the twist of conventional ring-spun yarns prevents the full utilisation of the fibre reinforcement potential in the composites [19]. The higher the yarn twist, the lower the mechanical properties of the composite [5,9,10]. Therefore, the staple fibre yarns should be produced without a twist, as described in [7]. For the research and development of composites reinforced with an alternative flax yarn without a twist, the processes described in Figure 1 were realised. The work involves the industrial carding of flax tow, spinning on a pilot plant scale, industrial weaving and processing into composite materials. The yarns will be processed into UD composites by pultrusion on a laboratory scale to evaluate the reinforcement potential. The fabrics are processed into composites by autoclave resin injection (vacuum injection process in an autoclave) and vacuum infusion to evaluate industrial processability. Compared to low-twisted yarns, high-twisted staple fibre yarns are more suitable for pultrusion processing. With low-twist yarns, the necessary force for the pultrusion process can often not be achieved due to the low strength of these yarns. However, pultrusion is an important process for producing unidirectionally reinforced continuous profiles [10]. The vacuum infusion process and the autoclave injection process are used for the industrial production of high-quality components. Both are relatively time-consuming processes. Autoclave resin injection generally results in a lower void volume, and a higher fibre volume fraction is possible in the composite [20,21]. The processes are carried out with two different resin systems, which are typically used by the companies to produce industrial components.

The projects’ ultimate goal is to produce a prototype of a leaf spring element for a bogie of a narrow-gauge railway, which is currently made of glass fibre-reinforced thermosets. With good mechanical properties, this component also requires high fatigue strength and good damping properties. Hybrid composites of flax and glass fibre are also being produced as part of the work, in addition to pure flax composites. Glass fabrics as the outer layers protect the flax fibres from external weathering, while the good damping properties of flax [22], as the inner layer, can lead to a beneficial combination of properties in the hybrid composite laminate.

It is known that natural fibres lead to good damping properties superior to glass fibres [22,23,24]. Additionally, flax fibre-reinforced composites generally have better sound absorption properties than glass fibre-reinforced composites [22,25]. The fatigue strength of flax and glass fibre-reinforced composites shows different behaviour. While glass fibre-reinforced composites have a higher static tensile strength than flax fibre-reinforced composites, the strength decreases significantly more with increasing load cycles. If a sufficient number of cycles is realised, it can be assumed that the fatigue strength of glass and flax fibre-reinforced composites does not differ significantly [26]. With a very high number of load cycles, it is also possible that flax fibre-reinforced composites have a higher fatigue strength. Gassan showed that the fatigue strength of flax fibre-reinforced composites is strongly dependent on the mechanical properties and the structure of the composite. It was possible to prove that a higher strength and stiffness of the fibres, a stronger fibre/matrix adhesion, and a higher fibre content led to higher critical loads for damage initiation and higher failure loads. The damage progression rates were reduced as well. A UD fibre-reinforcement was less sensitive to fatigue-induced damage than composites reinforced with plain fabrics [27]. Similar results were confirmed by Bensadoun et al. [26]. Asgarinia et al. [28] have shown that minimised yarn crimp is a major concern to in-crease the resistance to fatigue damage of flax-reinforced composites.

It has also been shown that hybridisation can have a positive effect on fatigue. Flax and basalt laminates (similar properties to glass fibres) were hybridised, and a better-normalised fatigue resistance was obtained for the hybrid composite compared to the pure basalt composite [29]. Various studies are available in which the favourable properties of combining different materials or types of fibres have been investigated. For example, flax and carbon [30,31,32,33,34], flax and basalt [29,35,36], flax and graphite [37] flax and Kevlar [38] and flax and glass [39,40,41,42,43,44,45,46,47,48,49,50] were hybridised. If glass as outer layers and a flax core are used in the hybrid material, water absorption can be significantly reduced compared to a pure flax composite [40,45].

Therefore the present study aims to develop a hybrid composite made of flax and glass fibres in addition to a cheaper staple fibre yarn made of flax, which should lead to good mechanical properties in the composite. The flax fibres are to be protected by outer layers of glass fibres.

On the one hand, this study will examine the reinforcing effect of the newly developed yarns and, on the other hand, the processability of the fabrics and hybrid laminates. The properties are compared with composite materials produced from a UD flax roving, available on the industrial market, and from a UD glass roving.

## 2. Materials and Methods

### 2.1. Materials

A Lincore flax fibre roving type FR 500 with a fineness of 500 tex and a density of 1.45 g/cm^3^ (Depestele, Bourguébus, France) and a glass fibre roving type EC16 200 TD22C with zero-twist and a fineness of 200 tex (Saint-Gobain Vetrotex, Aachen, Germany) were used as reference samples. The yarns developed in the project and the resulting fabrics and composite materials were made from field retted flax from a longitudinal fibre line of scutched tow and was provided by SachsenLeinen GmbH (Markkleeberg, Germany). As matrices, an epoxy resin (type Epikote RIMR 135 with hardener Epikure RIMH 137, Momentive Specialty Chemicals B.V., Rotterdam, The Netherlands; mixing ratio 100:30; density 1.15 g/cm^3^) and a bio-based epoxy resin PTP-L (Polymer Material Made of Triglycerides and polycarbon acid anhydrides; density 1.1 g/cm^3^), produced by company Biocomposites And More (B.A.M., Ipsheim, Germany), were used.

### 2.2. Fibre Analysis

Fibres/fibre bundles from all process stages (raw fibres, slivers, yarns and fabrics) were examined after conditioning in a standard climate, according to DIN EN ISO 139 [51].

Manually created length staples were used to assess the length distributions of the flax tow visually. For this purpose, the fibre bundles were conditioned for 18 h at 20 °C and 65% relative humidity in a climate chamber type VCL 4003 (Vötsch Industrietechnik GmbH, Reiskirchen-Lindenstruth, Germany) and then weighed at 0.6 g with a scale type Kern ABT 120-5 DM (d = 0.00001 g; Kern und Sohn GmbH, Balingen, Germany). Individual fibre bundles were then stuck to copying film with double-sided adhesive tape, arranged in order of ends, and scanned with an incident light scanner (Epson Perfection Photo V700, Epson, Meerbusch, Germany) at 600 dpi with a black background.

Tensile characteristics of the single fibre bundles were determined with a Fafegraph M testing machine (Textechno, Mönchengladbach, Germany), working with a pneumatic clamping system (PVC clamps) and a 10 N load cell. Per test series 80 single elements were investigated at a gauge length of 20 mm and a testing speed of 10 mm/min.

### 2.3. Fibre Processing, Yarn and Fabric Production

The fibre bales with the flax tow were opened and air-dried for 10 days for the card slivers’ production. At SachsenLeinen company (Markkleeberg, Germany), flax was further processed and stretched (Schlumberger GN 5, Paris, France) with a processing speed of 30 m/min on a carding machine (Russian design, UDSSR) into card slivers with a fineness of 4600–6400 tex. Tape-laying technology was used employing work cards, and needle tape draw frames. Some of the slivers were re-stretched once (re-stretched) or twice (2× re-stretched).

Yarns were produced with 30 m/min from the different slivers with a fineness of approx. 945 and 200 tex. Fibres in yarns are held together with a polyamide polyfil (78 dtex, 20 single endless fibres) that is spun helically around the fibre yarn, as described in [7].

Quasi-UD fabrics were manufactured in the weft with a warp system of polyester (Nm 50/2; Polyester Staple Fiber(SD OB); Chung-Shing Textile Co., Ltd. Yang-Mei Chemical Fiber Factory, Taipei, Taiwan) from the staple fibre yarns as well as the flax and glass reference rovings with a rapier weaving machine, type HTV S (Lindauer Dornier GmbH, Lindau, Germany). The weaving machine has a width of 180 cm and is equipped with an electronic dobby, electronic warp drain and electronic take-up motion. A weaving speed of 200 1/min and a weft insertion rate of 236 m/min with a weft density of 12 threads per centimetre was set on the machine. The fabric composition was determined by taking a 15 × 15 cm^2^ sample of each fabric and dividing it into warp and weft threads. For the dried state (drying for 18 h at 60 °C in an oven, type UN 450, Memmert GmbH & Co. KG, Schwabach, Germany), the fabrics mass per unit area and composition are listed in Table 1.

### 2.4. Composite Production

Composites were produced in 3 different ways: pultrusion, vacuum infusion and autoclave injection. Pultrusion was used to investigate the reinforcement potential of the newly developed staple fibre yarns. Autoclave injection and vacuum infusion were applied to analyse the industrial processing of developed fabrics.

Unidirectional composites in the form of round and rectangular rods were produced with epoxy resin using miniature pultrusion. The process is described in detail in [52]. The round rods have a length of 50 cm and a diameter of 5.5 to 5.8 mm. The fibre volume fraction was approx. 40%. The rectangular samples have a length of 50 mm, a width of 10 mm and a thickness of 4.5 to 4.7 mm with a fibre volume fraction around 30%. All rods were post-cured at 60 °C for 18 h in an oven (type UN 450, Memmert GmbH & Co. KG, Schwabach, Germany). The rods were cut to the required length for impact and bending tests.

**Figure 2:**
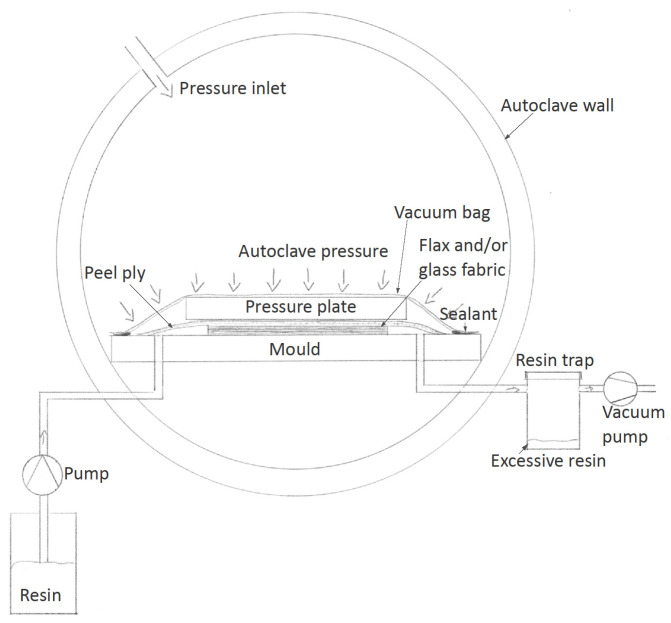
Schematic representation of the resin injection process in the autoclave (autoclave injection).

The quasi-UD fabrics were processed into composites with a PTP-L resin using a vacuum injection process in an autoclave (autoclave injection) (see Figure 2). The fabrics were dried in the autoclave before injection. For the vacuum injection process, an autoclave (type 33542, 2650 L, Scholz, Coesfeld, Germany) was used during pressure injection. Impregnation was carried out using a controlled vacuum with low differential external pressure on the moulds’ flexible top, which resulted in a relatively low fibre volume fraction. Subsequently, pressure and temperature were increased, and the composite was cured with a higher fibre volume fraction.

Furthermore, a vacuum infusion process was used with an epoxy matrix to form the quasi-UD fabrics into composites. Before processing, the flax fabric was dried for 16 h at 80 °C and was subsequently placed on a glass plate coated with a release agent. Peel ply and a flow aid were placed on top of this assembly. A spiral hose was used for filling with the epoxy resin and as a suction. A vacuum foil was applied, and the tightness of the vacuum was tested with a Greisinger GDH 200 measuring device (Regenstauf, Germany). The vacuum was generated with a vacuum pump type 20/2 from Vacmobiles (Auckland, New Zealand). The filling of the plates took 3–4 min each. Curing took place under ambient conditions. The composite plates from the vacuum infusion and autoclave injection process resulted in a thickness of approx. 2 mm and were cut by water jet cutting to the appropriate dimensions for further tests to determine the tensile, bending, impact and interlaminar shear strength (ILSS) properties. The characterisation of the specimens with regard to their mechanical properties took place at least three weeks after production.

Selected materials produced in the project are summarised in Table 2.

**Figure 3:**
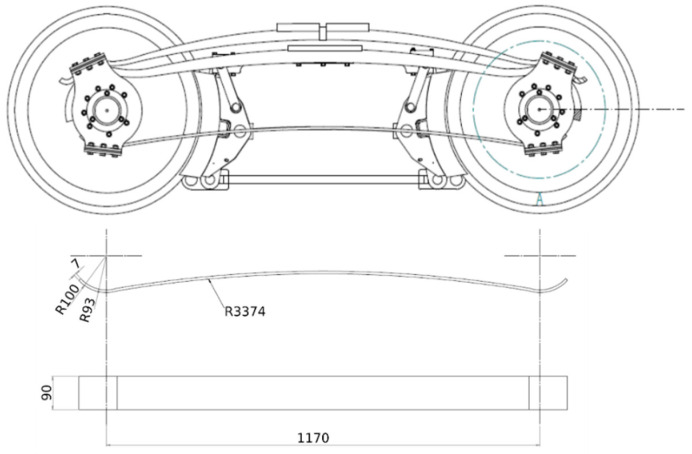
Leaf spring for a bogie of a narrow-gauge railway.

A leaf spring for a bogie of a narrow-gauge railway is being manufactured as a prototype (demonstrator) within the projects’ scope (see Figure 3). An RTM process (Resin Transfer Moulding) was used for the production. Three leaf springs are produced from woven fabrics of flax and glass and a hybrid of glass and flax. The fabrics were placed into a sub-mould which was coated with a release agent. The upper mould was placed on top, and both moulds were clamped as the resin and hardener mixture was added under pressure. After curing, the leaf spring was unmoulded. The demonstrator itself is only briefly presented in this publication and is the subject of ongoing investigations. It should be noted, at this point, that it was already possible to produce leaf springs from glass and flax fibre-reinforced thermosets. The focus is on developing and characterising the materials that will later be applied to the component.

### 2.5. Composite Characterisation

The composite materials were tested after climatic conditioning, according to the DIN EN ISO 291 standard [53].

Bending tests on 7 pultruded round rods (length 120 mm) were carried out according to DIN EN ISO 14125 [54], with a waisted load applicator (radius of waist: 6 mm) according to ISO 3597-1 [55] at a support distance of 96 mm. A pre-load of 10 N was applied. The testing speed was set to 5 mm/min. The test was carried out on a Zwick/Roell Z020 universal testing machine (Zwick GmbH and Co., Ulm, Germany) with a 20 kN load cell. The pultruded rectangular specimens with a length of 100 mm were also analysed, according to DIN EN ISO 14125 [54] at a span length of 74 mm. A testing speed of 1 mm/min was applied to determine the flexural modulus, which was afterwards accelerated to 5 mm/min on the Zwick/Roell universal testing machine. Five specimens were tested per test series. The bending tests of the composite laminates were carried out for 8 specimens each with a width of 15 mm and a length of 100 mm, following the DIN EN ISO 14125 standard [54] with a Zwick/Roell Z020 universal testing machine at a span length of 45 mm and a test speed of 1 mm/min. The flexural modulus of all bending test specimens was determined between 0.05% and 0.25% flexural strain via linear regression (deflection was calculated from the traverse path of the testing machine).

The interlaminar shear strength (ILSS) of the composite laminates was determined following standard 14130 [56] on 8 test specimens, each with a width of 10 mm and a length of 20 mm using the Zwick/Roell Z020 testing machine. The span length was set individually for each test series and resulted from 5-times the sample thickness at a 1 mm/min test speed.

Tensile tests of composite laminates of 8 specimens per test series, with a width of 25 mm and a length of 250 mm according to DIN EN ISO 527-2 [57], were carried out with the Zwick/Roell universal testing machine equipped with a 20 kN load cell and a Galdabini Quasar 250 universal testing machine (Galdabini (S.P.A.), Cardano Al Campo VA, Italy, load cell 250 kN) with a test speed of 2 mm/min. The Young’s moduli of all composite laminates were determined with the Zwick/Roell universal testing machine. The elongation was recorded with a video extensometer (VideoXtens, Zwick/Roell GmbH, Ulm, Germany) between 0.05% and 0.25% strain after reaching a pre-load of 50 N. The tensile tests of the flax-reinforced composites were performed with the Zwick/Roell universal testing machine at a testing speed of 2 mm/min until breakage. Due to the higher required forces, the glass fibre-reinforced composites and the hybrid materials were run to break on the Galdabini testing machine at a 2 mm/min testing speed.

The density of the pultrusion samples was determined gravimetrically and via the volume of the measured samples. The density of samples produced by vacuum infusion and autoclave injection were determined using a displacement method [58] with a micro-balance KERN ABT-120-5DM (Kern & Sohn GmbH, Balingen, Germany). For the investigations, commercially available canola oil with a density of 0.92 g/cm³ was used. 

## 3. Results and Discussion

### 3.1. Fibre Characteristics

**Figure 4:**
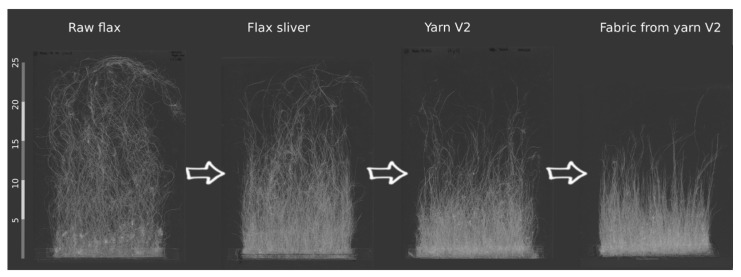
Length distribution depending on the subsequent processing—from raw flax to fabric.

**Figure 5:**
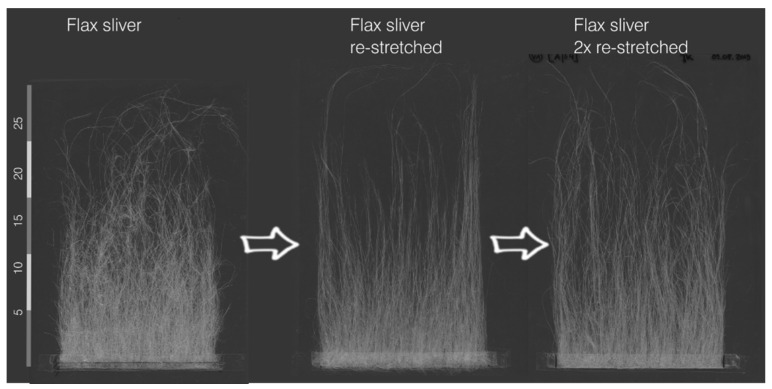
Length distribution depending on re-stretching of the sliver.

The results for determining the change in fibre bundle length during the process are shown as an example in Figure 4. Raw flax displays quite long and coarse fibre bundles. Due to the sliver production, a shortening of the fibre bundles is visible. However, many fibre bundles are split and become finer during carding. The yarn production shows a further slight shortening of the fibre bundles, whereby the length distribution becomes more and more homogeneous from process step to process step. No further significant shortening of the fibre bundles was observed due to the manufacture of the fabrics. The shortening during the process follows a similar trend for all yarns and fabrics produced. It can be shown that the re-stretching of the sliver does not lead to any significant shortening of the fibre bundles (compare Figure 5).

**Figure 6:**
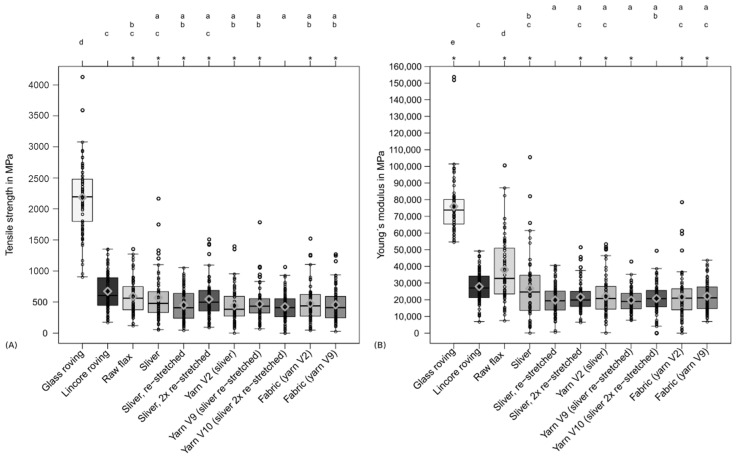
Properties of raw flax and single fibre bundles prepared from slivers, yarns and fabrics. (**A**) Tensile strength and (**B**) Young’s modulus shown as box-whisker plots with rhombuses as mean values. Different letters indicate significant differences between the results. Results that are not normally distributed are marked with *.

The tensile properties of individual fibre bundles taken from the various processing steps are presented compared to the fibres from Lincore and glass roving in Figure 6. The high scatter of the glass fibres results can be explained by the sample preparation and the defects that are probably introduced on the fibre surface. The tensile strength results of the selected raw flax are comparable to that of single fibre bundles from the Lincore roving (see Figure 6A). Due to the sliver production, there is a slight, non-significant reduction in tensile strength, and further process steps had no negative impact on the tensile strength from a statistical point of view. In contrast, Young’s modulus of the selected raw flax was significantly higher than the fibre bundles from the Lincore roving (see Figure 6B). However, the Young’s modulus was significantly reduced by further processing into a sliver and re-stretching it. The subsequent process steps of yarn and fabric production, on the other hand, had no negative influence on the tensile modulus.

### 3.2. Composite Characteristics

As described above, pultrusion samples were prepared to evaluate the fibres’ reinforcing effect or the developed yarns. Fabrics were processed by vacuum infusion and autoclave injection to test industrial processability.

### 3.3. Pultruded Composites

**Figure 7:**
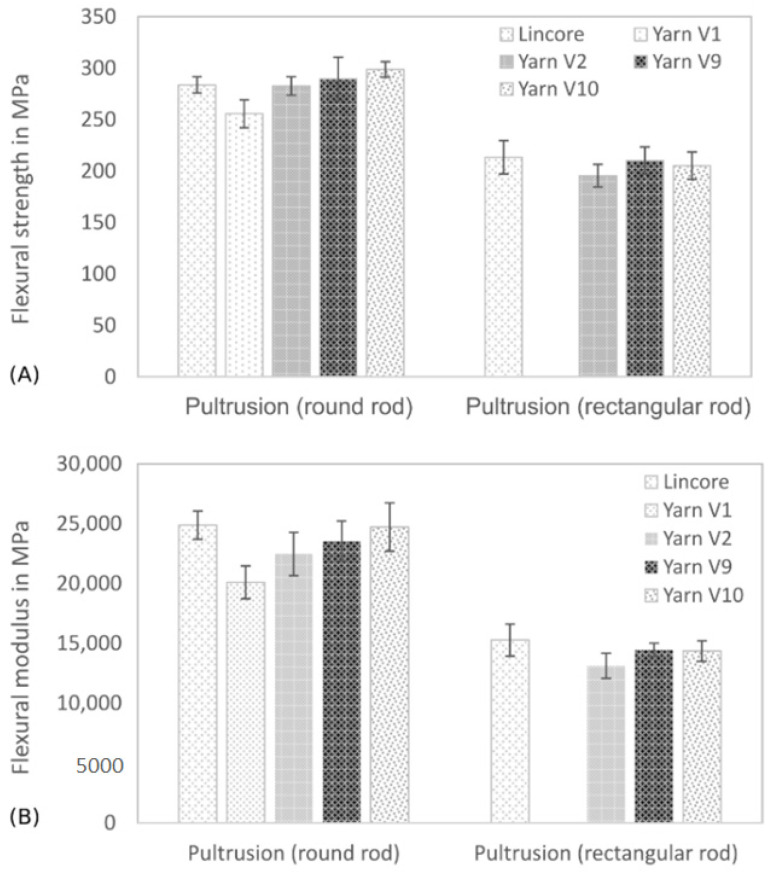
Flexural strength (**A**) and flexural modulus (**B**) of pultruded round and rectangular rods (fibre volume fraction of round rods ~40% and of rectangular rods ~30%). Results are shown as mean values with standard deviation as error bars.

The results of the flexural properties of round rods with a fibre volume fraction of ~40% and rectangular rods with a fibre volume fraction of ~30% are summarised in Figure 7. In the course of yarn development, a significant improvement of the reinforcing effect was achieved, which led to improved composite flexural properties. Similar trends were demonstrated for flexural strength (Figure 7A) and flexural modulus (Figure 7B). The aim was to achieve properties comparable to those of composites reinforced with the commercially available Lincore flax roving. The round bars’ results have shown that the bending properties with the first developed yarn (yarn V1, 945 tex) are significantly lower than the bending properties of the Lincore sample. In further trials, the yarn fineness was reduced considerably to 213 tex (yarn V2), and a significant increase in the bending properties was achieved, showing comparable values to the Lincore samples. However, the flexural modulus was significantly lower. Additional optimisations and developments further improved the yarn structure. Fibre undulations in the yarn were reduced (yarn V9, 200 tex) and a re-stretched sliver was used for yarn V9. The fibre bundles in the sliver did not differ significantly in length (compare Figure 5), but the sliver seemed to be more homogeneous and showed a higher fibre orientation. Both aspects had a positive effect on flexural strength and flexural modulus. From a statistical point of view, both characteristics are comparable to that of the Lincore reference sample. A second re-stretching of the sliver for yarn production (yarn V10, 200 tex) led to a further, but not significant, increase in the flexural properties.

The advanced yarns (V2, V9 and V10) were evaluated using the rectangular samples and compared to the Lincore reference sample. Overall, the rectangular rods show significantly lower flexural properties compared to the round samples. The results are mainly due to the differences in fibre volume content (rectangular bars between 30% and 32%; round bars between 42% and 46%) (see Table 3). The rectangular samples also have a higher porosity compared to the round bars. Voids can lead to defects, reduced fibre/matrix adhesion and, thus, to earlier failure. It was still possible to improve the bending properties with yarn V9 compared to yarn V2, and comparable properties with those of the Lincore reference sample were obtained. However, in this case, a double re-stretching of the sliver (yarn V10) did not result in a further improvement of the bending properties. The properties are comparable to the composites reinforced with yarn V9. A slightly increased void volume (see Table 3) may lead to a different trend compared to the round rods. For comparison purposes, the glass roving was pultruded into a round rod with a fibre volume content of 46.5% resulting in flexural strength of 429 ± 115 MPa and a flexural modulus of 26.5 ± 3.4 GPa.

The lightweight potential of materials can be estimated by dividing the mechanical properties by the density. In that case, the glass composite has a specific flexural strength of 246 MPa/(g·cm^−3^) and a specific flexural modulus of 15.2 GPa/(g·cm^−3^). In comparison, the composite reinforced with yarn V9 achieved a specific flexural strength of 224 MPa/(g·cm^−3^) and a specific flexural modulus of 18.2 GPa/(g·cm^−3^). Based on this consideration, the specific stiffness of the flax composite is even higher than that of the glass-fibre-reinforced material. By increasing the fibre volume fraction of yarn V9 to 55%, a specific flexural strength of 244 MPa/(g·cm^−3^) and a specific flexural modulus of 20.9 GPa/(g·cm^−3^) at lower density (see Table 3) is achieved.

Good mechanical properties were obtained for the round bars (fibre volume fraction ~43%) with a maximum flexural strength of 290 MPa and a flexural modulus of 23.5 GPa, and for the rectangular specimens (fibre volume fraction ~32%) with maximum flexural strength and flexural modulus of 210 and 14.5 GPa, respectively. A flexural strength of 322 MPa and a bending modulus of 27.6 GPa was achieved for round rods with a fibre volume ratio of 55%. For comparison, the literature measures, for example, flexural strength values of 190 MPa for a UD composite reinforced with yarn (fibre volume fraction 28%) [10], 218 MPa for a UD composite with a flax sliver (fibre volume fraction 40%) [59] or 230 MPa for a thermoplastic composite produced in a pultrusion process (fibre volume fraction 50%) [60]. The previously mentioned yarn-reinforced composite reached a flexural modulus of 16 GPa [10], and that reinforced with the sliver a value of 17.7 GPa [59].

**Figure 8:**
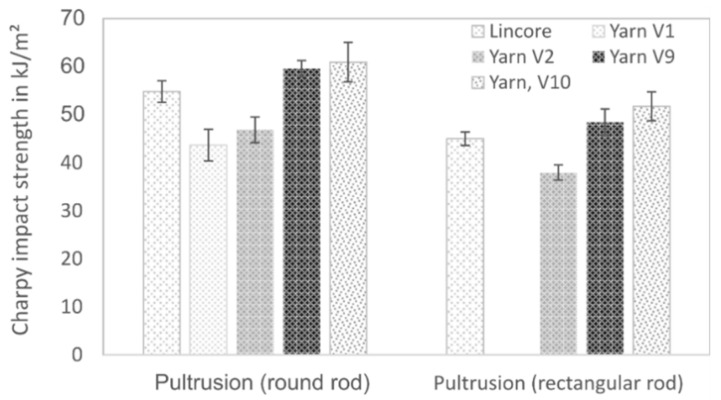
Unnotched Charpy impact strength of pultruded round and rectangular rods (fibre volume fraction of round rods ~40% and of rectangular rods ~30%). Results are shown as mean values with standard deviation as error bars.

The pultruded samples’ impact properties show a similar trend for the round samples, as described for the flexural properties (Figure 8). The further development of the coarse (945 dtex) yarn V1 to the finer 200 tex yarn (yarn V9 and V10) led to a significant improvement in impact strength. In contrast to the flexural characteristics, the impact strength could even be exceeded compared to the Lincore reference sample. The same trend was found for the rectangular samples, although the values are lower due to the lower fibre volume fraction. An additional effect could be the different test specimen geometry (round versus rectangular). Again, the impact strength of the Lincore reference sample was exceeded using the yarns V9 and V10. Concerning impact strength, the flax samples show apparent weaknesses compared to the glass fibre-reinforced sample with a value of 394 ± 20 kJ/m^2^ (round bar). The maximum values of the round rods (fibre volume fraction 43%) are comparable with a thermoplastic composite material containing 50% pultruded flax fibres (60 kJ/m^2^) [60]. Increasing the fibre volume fraction to 55% in a round sample of yarn V9 resulted in a value of 91 ± 3 kJ/m^2^. This value is relatively high for bast fibre-reinforced plastic. The literature shows impact strength values in the range of 14–20 kJ/m^2^ for bast fibre-reinforced epoxy (fibre mass fraction 65%) and between 25 and 35 kJ/m^2^ for bast fibre-reinforced polypropylene (fibre mass fraction 50%) [61].

In summary, the goal was reached to obtain comparable mechanical properties to high-quality scutched long flax in a composite material by using staple fibre yarn. The previous statement is valid for the bending properties; for the impact behaviour, the Lincore roving-reinforced composite properties could even be outperformed.

### 3.4. Composite Laminates from Fabrics

The fabrics made from yarns were processed by vacuum infusion and autoclave injection to test the processability and mechanical composite properties. A standard epoxy resin was used for the composites produced by vacuum infusion, while a bio-based epoxy matrix (PTP-L) was used for the composites produced by autoclave injection. Additionally, hybrid materials made of flax and glass fabrics were produced to assess the composites’ suitability for use in a leaf spring of a narrow-gauge railway bogie. This hybridisation will be used to combine the favourable properties of both types of fibres (good resistance to environmental influences of glass and good damping properties of flax). For comparison, a Lincore and a glass fabric were produced in the same manner into composites.

The results of the composite materials from the different manufacturing processes are not directly comparable, since different numbers of plies were used and the processes resulted in different fibre volume fractions. Table 4 shows the number of fabric plies used and the measured density and theoretically calculated volume fraction of PES warp threads and reinforcing fibres in the materials. By processing in an autoclave, higher volume fractions could be realised compared to vacuum infusion.

**Figure 9:**
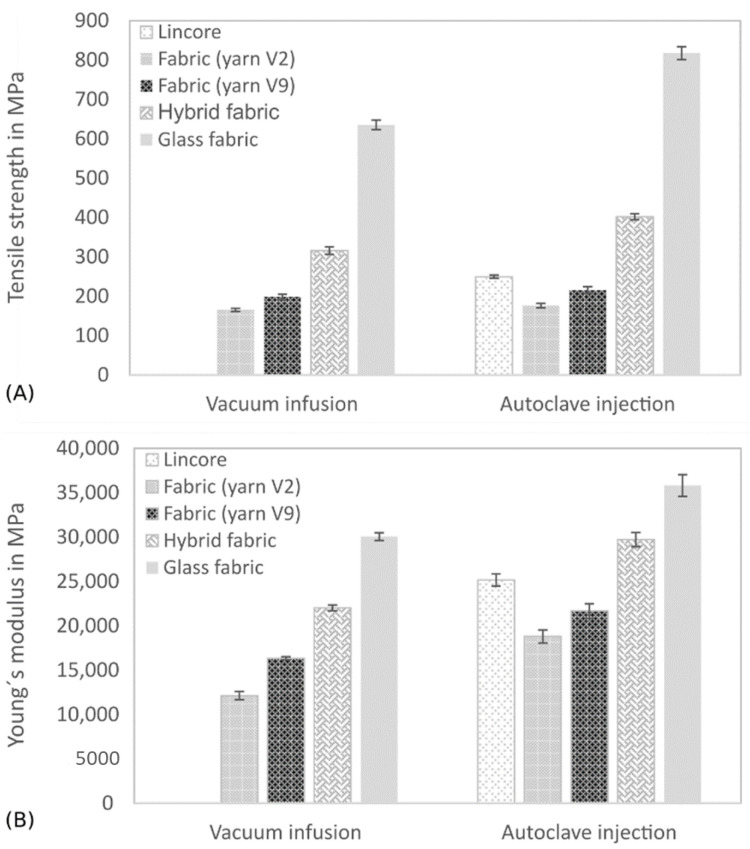
Tensile strength (**A**) and Young’s modulus (**B**) of composites produced by vacuum infusion and autoclave injection. The results are shown as mean values with the standard deviation as error bars.

The results of the tensile properties of composites produced in two different ways show a similar trend despite a different fibre volume fraction (compare Table 5 and Figure 9). However, the Lincore fabric was only processed using the autoclave injection method.

**Figure 10:**
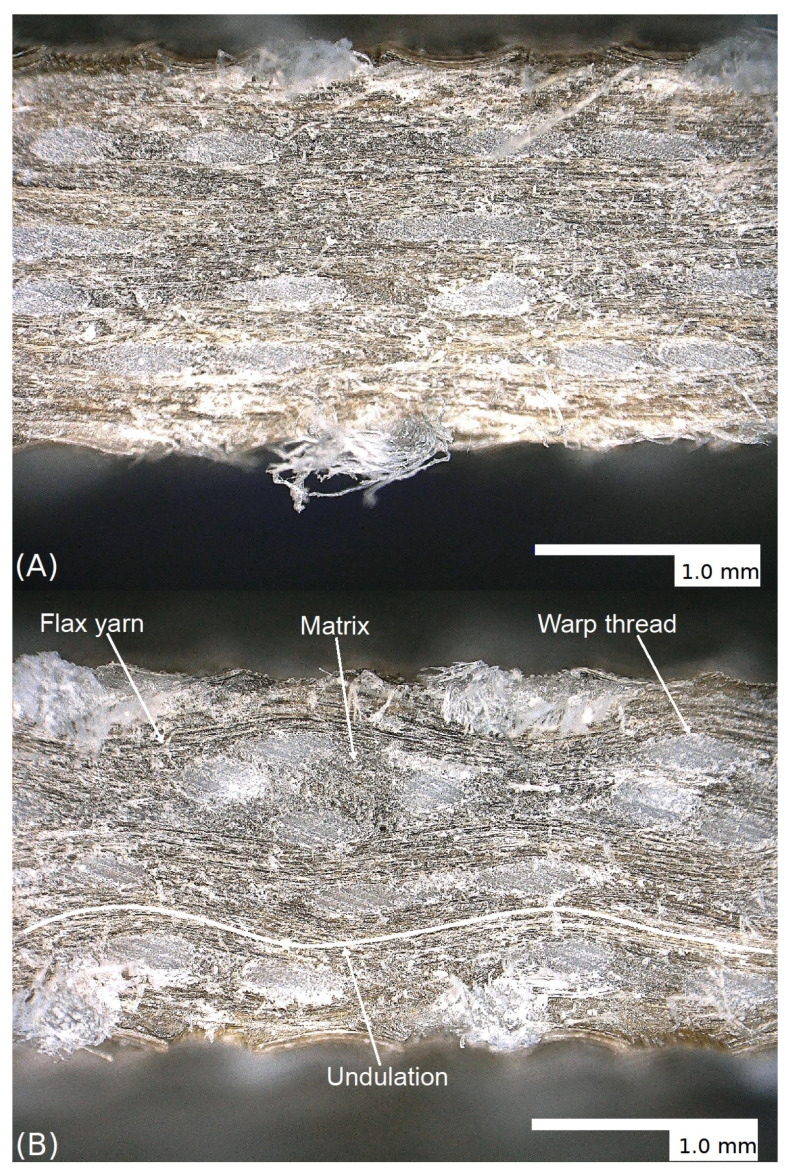
Micrographs (Keyence digital microscope VHX 6000, Osaka, Japan) of **(A)** composite laminates manufactured from the Lincore fabric and **(B)** a flax fabric (yarn V9) in a longitudinal direction.

Both methods have shown a significant improvement in tensile strength (Figure 9A) and Young’s modulus (Figure 9B) due to the further development of yarn V2 to yarn V9. The tensile strength and Young’s modulus of the Lincore laminate were not reached, in contrast to the pultrusion samples, where the samples with yarn V9 showed comparable results to the samples with the Lincore roving. As the fibre volume fraction does not differ significantly from the Lincore laminate with 48.0% and the flax fabric (yarn V9) laminate with 50.7%, the question arose whether fibre orientation differs due to fabric production. This assumption was confirmed by the microscopic examination of longitudinal sections of the samples. Figure 10 illustrates that the Lincore roving (Figure 10A) is almost unidirectionally oriented in the fabric, while the yarn V9 (Figure 10B) shows an undulation in the fabric. It is proven in the literature that a fibre orientation deviating from the load direction leads to a considerable reduction in strength and stiffness in the composite [5,9,10]. There is some evidence to suggest that this effect may be due to the more circular cross-section of the yarn compared to the flat Lincore roving. In order to verify which effect is responsible for the differences, UD laminates were produced in a winding process (data not shown). The laminates’ results with yarn V2 already show comparable tensile characteristics to the Lincore laminate produced by winding. Therefore, it is assumed that the undulation in the fabric is mainly responsible for the lower tensile properties. This undulation will be minimised in the future optimisation of the weaving process.

In order to classify the mechanical properties of the flax fibre-reinforced composites with state of the art, results are compared with that of similar composites from the literature and are summarised in Table 6. The comparison proves that the tensile properties of the materials made from the staple fibre yarns are similar to the high-performance composites described in the literature. Due to the higher fibre volume fraction in the autoclave injection process, composites reinforced with glass fibres show better tensile properties than materials produced in the vacuum infusion process. A hybridisation of glass and flax fabrics (outer glass layers and inner flax layers) leads to a significant improvement in the tensile properties, compared to the flax composites, as already described by other studies [40,41,42,45,48,50,62]. Again, the characteristic values of the samples processed in the autoclave injection process are significantly higher. In addition to the higher fibre volume fraction, this effect is also based on the fact that only two glass layers and three flax layers were processed in the vacuum-infusion process and a total of four glass layers and three flax layers in the autoclave injection process. This statement can also be supported by a study by Saidane et al. [45]. The authors investigated an increasing number of outer glass layers (2 to 8) in a hybrid material with flax and could demonstrate a continuous increase in tensile strength and tensile modulus.

**Figure 11:**
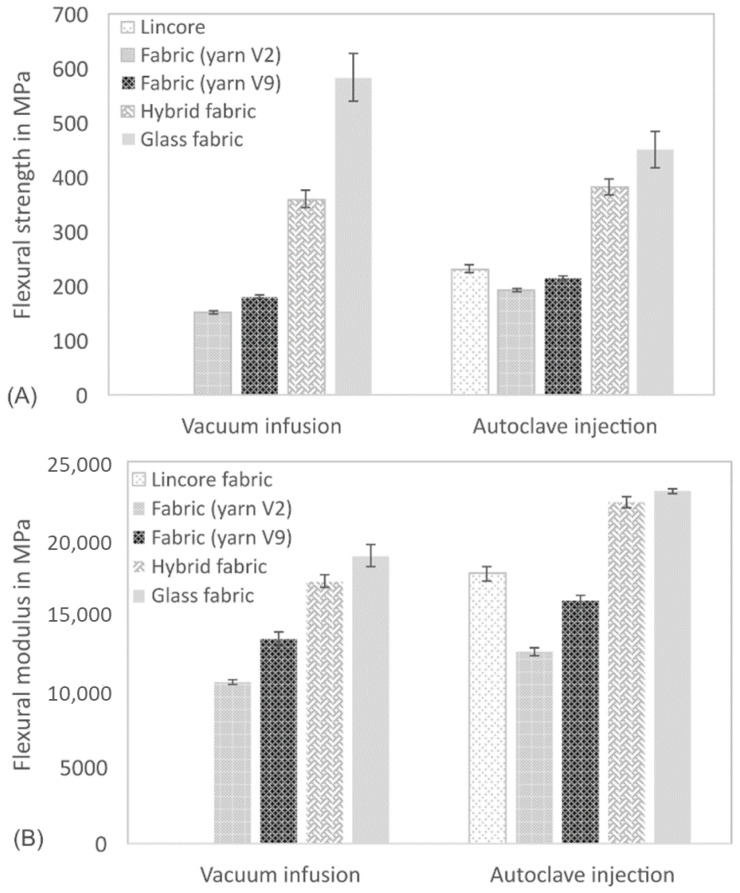
Flexural strength (**A**) and flexural modulus (**B**) of composites produced by vacuum infusion and autoclave injection. Results are shown as mean values with standard deviation as error bars.

**Figure 12:**
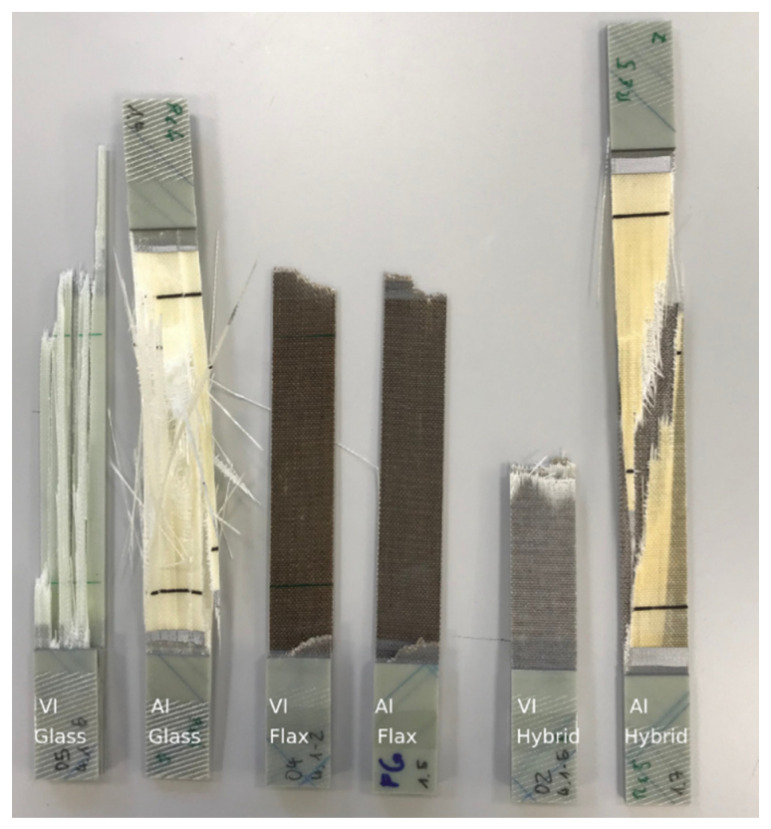
The appearance of fracture after tensile tests of composite laminates produced by vacuum infusion and autoclave injection (VI = vacuum infusion, AI = autoclave injection).

**Figure 13:**
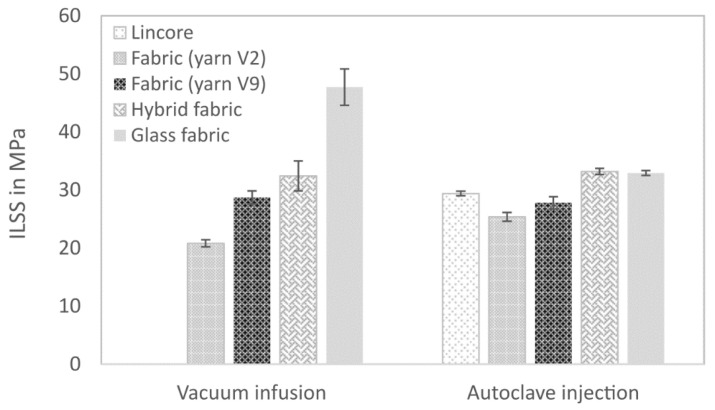
Interlaminar shear strength (ILSS) of composites produced by vacuum infusion and autoclave injection. Results are shown as mean values with standard deviation as error bars.

The results of the bending tests are summarised in Figure 11 and Table 5. Except for the glass fibre-reinforced composite, produced with the autoclave injection process, the results’ trend is similar to that of the tensile tests, but with different degrees of intensity. The further development of the V2 to V9 yarn has improved the flexural properties of the composites. As demonstrated in the tensile tests, the reinforcing effect of the Lincore fabric could not be achieved yet. As previously discussed, this is caused by the stronger undulation of the yarn in the fabric (compare Figure 10). The flexural modulus (Figure 11B) of the glass fibre-reinforced composite produced by the autoclave injection process is significantly higher than that of the material produced by the vacuum infusion process for the reasons mentioned above, whereas the flexural strength shows the opposite trend (Figure 11A). The materials from the two different manufacturing processes show considerable differences in failure with regard to fracture behaviour under tensile testing (Figure 12). While the samples from the vacuum infusion process failed—they were brittle and formed distinct cracks in the fibre direction—the sample from the autoclave injection process showed distinct delamination, which probably caused an earlier failure. The samples produced have a thickness of 1.7–2.1 mm. The maximum difference in the thickness of samples prepared from the same fabrics by vacuum infusion and autoclave injection is 0.3 mm. However, in flax, five layers were used for autoclave injection and only four layers for vacuum infusion, leading to significant differences in fibre volume fraction (36.5% for vacuum infusion and 50.7% for autoclave injection; compare Table 4). The glass fibre-reinforced composite produced by autoclave injection has a fibre volume content of 42.7%, which is significantly higher than that of the composite produced by vacuum infusion (30.5%). The same is true for the hybrid composites, which show a fibre volume fraction of 47.4% for the samples produced by autoclave injection and 34.4% for samples manufactured with the vacuum infusion process. It has to be taken into account that the samples from the autoclave injection process were produced with a total of four glass layers and those from the vacuum infusion process with two glass layers (compare Table 4). When interpreting the results, it must be taken into account that while the fabrics used and the fibres’ orientation are identical in the composites produced with different processes, the resin systems differ. Compston and Jar [66] found that the mode I interlaminar fracture toughness of glass fibre-reinforced vinylester is not affected by the fibre volume fraction in the range of 32–52%. Budan et al. describe, for drilling hole experiments in glass fibre-reinforced plastics, that delamination increases significantly with increasing fibre volume fraction from 30% to 70% [67] which may be a reason for the higher delamination of the autoclave injection samples due to higher fibre load. Additionally, it is assumed that the manufacturing process and different matrices also contribute to different fracture behaviour. It is well known that the quality of the interface between the matrix and the fibres has a strong effect on the composite mechanical properties, especially on the strength values [68]. Since the flexural modulus is determined in the initial slope of the stress–strain curve at relatively low-stress values, the influence of fibre/matrix adhesion is less than on strength. As a measure of fibre/matrix adhesion, short beam shear tests were carried out to determine the interlaminar shear strength (ILSS). The results confirm a lower fibre/matrix adhesion of the glass fibre-reinforced composite produced by autoclave injection (Figure 13, Table 5). Due to the quasi-UD continuous fibre reinforcement, the influence of the lower interfacial properties under tensile load is smaller compared to the bending test. In the bending test, a lower ILSS leads to a significant reduction in bending strength. Production in an autoclave usually leads to better-compacted materials with lower void volume and higher fibre volume fraction [20,21], which should positively influence the fibre/matrix adhesion. However, this effect cannot generally be demonstrated by ILSS determined with the short beam shear test. Despite this aspect, the ILSS shows comparable results for the flax laminate (yarn V9) and the hybrid material produced with different processing methods. It has to be considered that the fibre volume fraction of the materials produced by autoclave injection is significantly higher. However, Esnaola et al. [69] found that the fibre volume fraction of glass fibre-reinforced epoxy has no significant effect on the ILSS as determined by the short beam shear test. Nevertheless, a higher void content in the vacuum infusion samples is expected to have a negative effect on the ILSS due to the reduction in the sliding resistance between individual fibre layers by an increasing void content [70]. For the results presented here, it is assumed that the epoxy resin used in vacuum infusion leads to better fibre/matrix adhesion than the bio-based epoxy resin used in the autoclave injection process. However, this hypothesis needs to be verified in future work. The influence of voids greatly impacts the transverse tensile properties of UD materials, especially the transverse tensile strength [71]. The interface debonding load should be analysed by transverse tensile tests, as described by Zhou et al. [72], in future research.

The hybrid materials show significantly better flexural properties than the flax laminates, which various authors also described [40,41,42,48,62]. These authors showed that the presence of external layers of glass fabric in a flax/epoxy laminate positively influences the mechanical properties. In our study, especially the flexural modulus could be remarkably increased and was closer to the glass fibre-reinforced composite than to the flax fibre composite (Figure 7B). This trend regarding bending properties is more pronounced than the tensile modulus (Figure 9B) and can be explained by the loading direction. During the bending test, glass is located as the outer layer in the hybrid material and is loaded mainly on the tensile side, whereas the entire specimen cross-section is stressed in the case of tensile loading. This effect was described by Selver et al. [48] for flax/glass hybrid composites and is also reported for plastics with layered cellulose fibre-reinforced composites (compare, e.g., [73,74]). Selver et al. [48] found that a changing stacking sequence did not significantly affect the tensile strength and modulus of composites, whereas there were notable differences in composites’ flexural strength when the outer layers contained glass fibres. The flexural modulus of this material was comparable to that of the pure glass fibre-reinforced composite. When using flax as outer layers, the flexural modulus and flexural strength were considerably lower. Related to this, Jusoh et al. report that the tensile properties are less influenced by the stacking order than the flexural properties [42]. Since the flexural modulus’ determination takes place in the linear-elastic region of the stress-deflection curve, it is assumed that the influence of the outer glass layers is stronger under bending load. The hybrid composite with a total of four glass layers produced in the autoclave injection process has comparable flexural strength to the hybrid composite made by vacuum infusion (Figure 7A). Similarly, as described for the glass fibre-reinforced composite, this behaviour of the hybrid composite may be due to the different failure that has occurred. As shown in Figure 12, the composite from the autoclave injection process shows clear delaminations between the glass and the flax layers, while the material produced by the vacuum infusion process shows hardly any delaminations. This conclusion is supported by the ILSS test, which shows comparable values for the materials produced by the autoclave injection and vacuum infusion processes (Figure 13).

**Figure 14:**
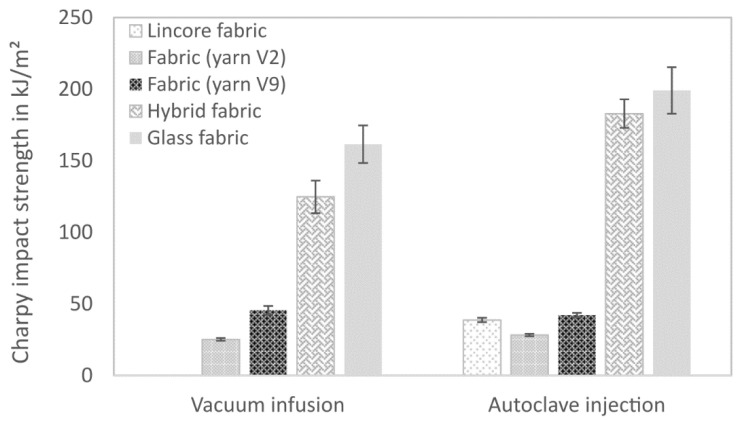
Unnotched Charpy impact strength of composites produced by vacuum infusion and autoclave injection. Results are shown as mean values with standard deviation as error bars.

The experiments on the unnotched Charpy impact strength prove that the further development of yarn V2 to V9 has led to a significant toughness improvement (Figure 14, Table 5). Although the fibre volume fractions differed (Table 4), comparable characteristic values were measured for the flax fibre-reinforced composite from the vacuum infusion process and the autoclave injection process. The impact strength of the V9 yarn-based fabric laminates (42 kJ/m^2^) is comparable to the laminates made from the Lincore fabric (39 kJ/m^2^). Undulation in the fabric seems to have a minor influence on the impact strength. Liu et al. [11] stated that toughness is dominated by the fibre volume fraction, fibre length and fineness rather than the reinforcement architecture.

The glass fibre-reinforced composite from the autoclave injection process has the highest impact strength. While delamination and reduced ILSS have a negative effect on the flexural strength, in case of impact stress, delamination can lead to increased energy absorption, and thus, to higher characteristic values [75]. Furthermore, the impact strength is positively influenced by the higher fibre volume fraction. The hybrid material from the autoclave injection process also shows significantly higher values than the vacuum infusion produced composites. The higher energy absorption due to delamination can also explain this (Figure 12) as well as the higher number of glass layers in the composite and a higher fibre volume fraction. The impact strength of both hybrid composites is significantly closer to that of the glass fibre-reinforced composite than to that of the flax fibre-reinforced composite (Figure 14). Papa et al. [35] determined a higher impact strength for a flax/basalt hybrid material than for the measured individual components. The authors justified their statement with the impact performance, characterised by the maximum impact stress value. The outer basalt layer resulted in the highest energy absorption with less delamination.

**Figure 15:**
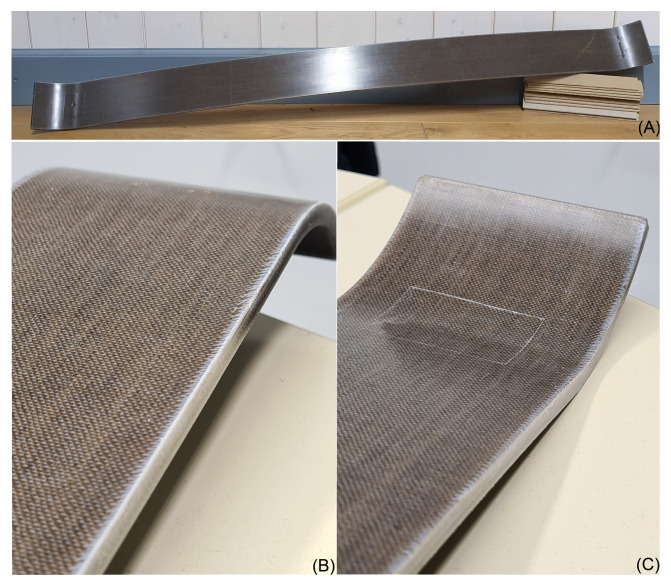
Prototype of a flax fibre-reinforced leaf spring of a bogie of a narrow-gauge railway produced by RTM, (**A**) overview, (**B**,**C**) detailed images (width of the leaf spring: 90 mm, length: 1170 mm).

In summary, the hybridisation of flax and glass was successful. For most of the mechanical properties, the characteristic values are close to those of the glass fibre-reinforced composites, especially under bending and impact stress. These two properties are essential for a leaf spring element, which is exemplarily shown as a demonstrator in Figure 15 made of the developed flax fabric. It is expected that hybridisation will also improve the thermomechanical properties, as demonstrated by the hybridisation of flax and carbon [30,34] or cellulose and basalt [52], and reduce water absorption or swelling [40]. Based on the work of Selver et al., it is considered that the improved damping of the hybrid material will result from the presence of flax compared to a pure glass fibre-reinforced composite. The authors determined the damping by dynamic mechanical analysis and found that natural fibre-reinforced composites and some of the hybrid composites had higher damping characteristics than glass fibre-reinforced composites [48].

Furthermore, the fabrics made of the newly developed staple fibre yarn show good mechanical properties. The investigations on pultruded samples show that the newly developed yarns’ reinforcing effect is comparable to a high-quality Lincore roving. Martin et al.´s [16] statement that well-prepared flax tow can lead to comparable mechanical characteristic values as high-quality scutched flax can be confirmed. In order to enhance the mechanical properties of the fabric laminates, it is necessary to reduce the undulation in the fabric. Based on such an optimisation of the process, it is expected that the tensile and flexural properties of the laminates will be equivalent to those of Lincore fabric laminates.

### 3.5. Lightweight Construction Potential

**Figure 16:**
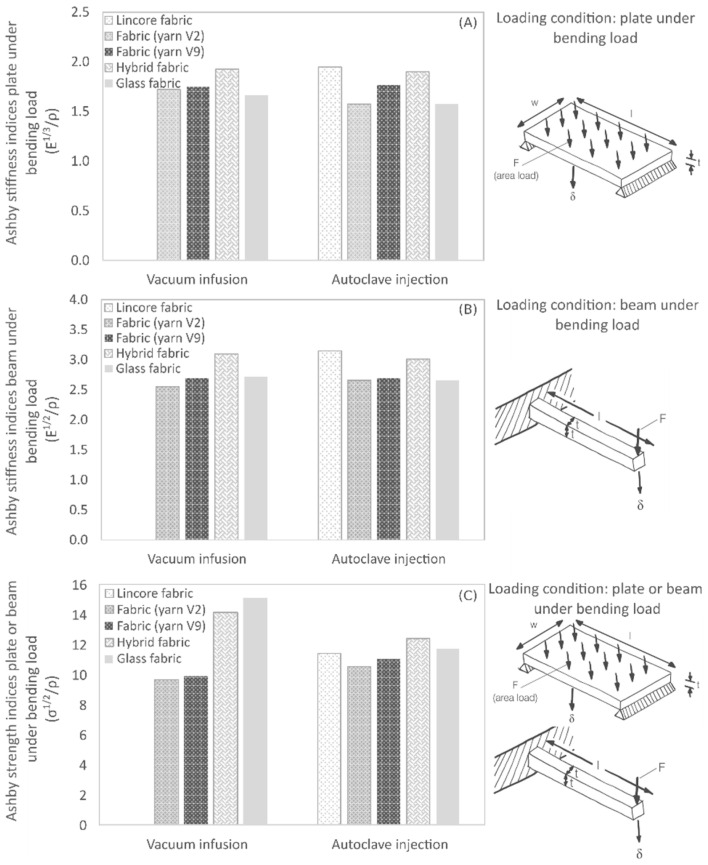
Ashby indices of composite materials produced by vacuum infusion and autoclave injection. The fibre orientation was taken into account in the longitudinal direction. (**A**) plate loaded in bending (stiffness-limited design at minimum mass; stiffness, length, width specified, thickness free), (**B**) beam loaded in bending (stiffness-limited design at minimum mass; stiffness, length, shape specified, section area free) and (**C**) plate loaded in bending (strength-limited design at minimum mass; stiffness, length, width specified, thickness free) or beam loaded in bending (strength limited design at minimum mass; load, length width specified, thickness free).

So-called Ashby indices were used to assess the lightweight potential of the composite materials. Under this approach, properties such as strength, stiffness, toughness or density are set in relation to each other in different ways. These performance indices can be used to identify the most suitable material for a specific load case. The material with the highest index value is ultimately the most suitable [76]. For example, if we assume that the composite laminates are to be used as a panel under bending load, the Ashby performance index (E^1/3^/ρ) is used (stiffness-limited design at minimum mass; objective: minimum mass; constraint: stiffness prescribed). E is the flexural modulus in GPa and ρ the density in g/cm^3^. In this case, the composite plates’ stiffness, length, and width are specified, while the thickness is free selectable. For the composites produced, the resulting performance indices are summarised in Figure 16A. For this load case and the specified optimisation objective, the laminate made of Lincore fabric and the hybrid materials made of flax and glass fabric show the highest indices. The index (E^1/3^/ρ) is also valid for a flat plate, compressed in-plane (buckling failure). This case is also relevant for the load on a leaf spring. For this optimisation objective, collapse load, length and width are specified, whereas the composite thickness is free selectable. The results show that the hybrid material offers advantages rather than disadvantages, compared to a conventional glass fibre-reinforced leaf spring. Comparable results can be found when the developed materials are to be used as beams under bending load (Figure 16B). The Ashby performance index, in this case, is (E^1/2^/ρ). Lincore fabric materials and hybrid materials also prove to be particularly suitable for this load, considering the potential for lightweight construction. The index (σ^1/2^/ρ) is valid for a flat plate loaded in bending (strength-limited design at minimum mass; objective: minimum mass; constraint: strength prescribed). The index (σ^1/2^/ρ) is as well used for a beam under bending load (strength-limited design at minimum mass; objective: minimum mass; constraint: strength prescribed/load, length and width are specified; thickness is free selectable), where σ is the bending (failure) strength and ρ the density. The described load case for the plate also corresponds to a load case that can occur with a leaf spring. Figure 16C illustrates that the hybrid composite also performs well for this load case (σ^1/2^/ρ). Glass fibre-reinforced composites still tend to offer a slight advantage over hybrid materials in terms of strength. The lower performance index of glass fibre-reinforced composites produced by autoclave injection is mainly due to their significantly lower bending strength (Figure 11A) and ILSS values (Figure 13). However, in terms of flexural strength, hybrid materials perform better. The flax laminates have lower mechanical characteristics compared to the reference laminate made of the Lincore fabric, due to the higher undulation of the staple fibre yarns in the fabric. Nevertheless, the reinforcing effect of the yarns was demonstrated in the pultrusion process. The performance indices of pultruded rectangular Lincore samples were the same as the composites reinforced with staple fibre yarns with values of 2.1 GPa^1/3^ (g cm^−3^)^−1^ for E^1/3^/ρ, 3.3 GPa^1/2^ (g cm^−3^)^−1^ for E^1/2^/ρ and 12.4 MPa^1/2^ (g cm^−3^)^−1^ for σ^1/2^/ρ. It is expected that a reduction in undulation in the fabric will also allow the laminates made from flax staple fibre yarns to have comparable performance indices to the Lincore laminate. The fabric laminates’ values are slightly lower than those stated for the pultruded UD samples due to the quasi-UD reinforcement (Figure 16).

Compared to a UD glass fibre-reinforced composite (fibre volume fraction 58%) with indices of 3.5 GPa^1/2^ (g cm^−3^)^−1^ for E^1/2^/ρ and 1.8 GPa^1/3^ (g cm^−3^)^−1^ for E^1/3^/ρ [77], the quasi-UD glass fibre laminates with 2.7 GPa^1/2^ (g cm^−3^)^−1^ for E^1/2^/ρ and 1.7 GPa^1/3^ (g cm^−3^)^−1^ for E^1/3^/ρ are slightly lower. In comparison, high strength steel has a value of 1.8 GPa^1/2^ (g cm^−3^)^−1^ for E^1/2^/ρ and 0.76 GPa^1/3^ (g cm^−3^)^−1^ for E^1/3^/ρ and an aluminium alloy of 3.0 GPa^1/2^ (g cm^−3^)^−1^ for E^1/2^/ρ and 1.5 GPa^1/3^ (g cm^−3^)^−1^ for E^1/3^/ρ [77].

In summary, all composites investigated have a significant advantage in terms of lightweight construction potential. In the overall view, the hybrid materials have the most suitable properties for achieving the maximum stiffness and strength for developing a leaf spring element. In the case of the hybrid material produced by the vacuum infusion process, it has a mass per unit area that is 12% lower than that of the glass laminate. The hybrid material produced by the autoclave injection process is even 20% lighter than the glass laminate.

Finally, it should be noted that a rough price estimation has shown that the costs for the semi-finished products made of flax could be significantly reduced by using flax tow. It is estimated that the price of high-performance flax fabrics can be reduced from around EUR 4.45–5.00 per m^2^ fabric when using cheaper flax tow for alternative yarns to around EUR 4.03–4.20 per m^2^ fabric. This is primarily due to the reduction in the raw material price from approx. 4.40 EUR/kg for high-quality long flax to approx. 1.00–1.50 EUR/kg for flax tow.

## 4. Conclusions

A nearly twistless staple fibre yarn was developed from flax tow for use in fibre-reinforced composites. Natural fibre-reinforced plastics with good mechanical properties currently require yarns with long fibre bundles of very high-quality, which is reflected in the high price. High-quality semi-finished products like fabrics made of long flax are almost on a par with carbon fibre semi-finished products. Nevertheless, developments with long flax yarns for structural components have already been accepted and established in the high-price segment. The staple fibre yarn based on shorter and less expensive flax tow offers the potential to overcome this deficit. The fibre bundles were only slightly damaged during processing. No significant reduction in the mechanical properties was observed from the sliver to fabric production.

**Figure 17:**
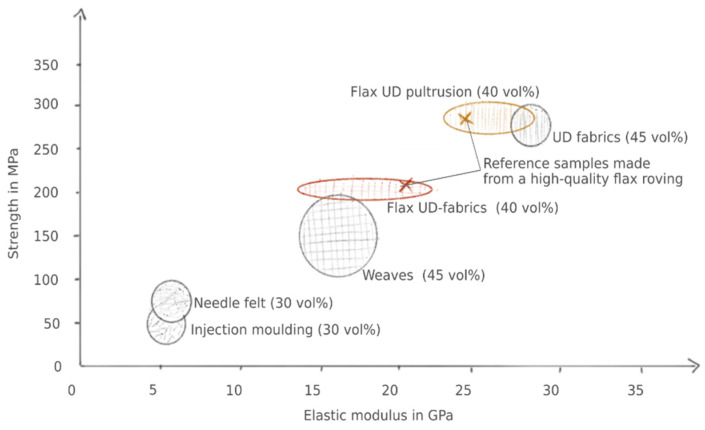
Classification of the mechanical characteristics of the produced composites compared to literature values and the reference composites.

The yarns’ reinforcing potential was proven with UD fibre-reinforced round, and rectangular rods produced by pultrusion and is comparable to that of a long flax roving available at the market (see Figure 17). The yarns were used to produce quasi-UD fabrics for the production of composite laminates by vacuum infusion or autoclave injection. The composite laminates were investigated concerning their tensile, bending and impact properties. The development of the staple fibre yarns and the improvement of the process parameters led to a significant increase in both the pultrusion samples’ mechanical properties and the laminates made from the fabrics. Other than the pultrusion samples, the bending and tensile properties of the fabric laminates could not yet reach the properties of the laminates from the long flax roving (Figure 17). Investigations showed that the staple fibre yarns’ undulation occurs in the fabric, which was not present in the long flax roving. The lower characteristic values are mainly attributed to the reorientation of the fibres. It is expected that comparable properties will be achieved if the undulation is minimised. It should be highlighted that the newly developed yarns led to an improvement in impact strength.

Hybrid materials with outer glass layers and inner flax layers are currently being produced for the leaf spring for a bogie of a narrow-gauge railway. The glass layers protect the flax fibres from environmental influences and positively affect strength and stiffness, whereas the flax fibres should provide improved damping compared to a component made of pure glass fibres. The hybrid materials have excellent mechanical properties and show clear advantages over a pure glass fibre-reinforced material in lightweight construction potential, particularly in flexural stiffness. Fatigue tests of the developed laminates are currently being carried out, and the leaf springs will be characterised in terms of their mechanical properties in future research.

Therefore, the less expensive staple fibre yarns from flax tow can be an alternative to high-priced long flax for the production of more highly-stressed components, such as a leaf spring. To classify the state of development of the staple fibre yarn in a Technology Readiness Level (TRL; 9 levels describing the product’s maturity), we would classify the stage of development of our textile product as level 6–7. Raw flax can be processed into slivers on an industrial scale, and industrial partners are already producing the fabrics and composites. The bottleneck currently exists in the spinning process. The yarns are produced in a pilot plant scale. Once, a spinning company is found that can produce the yarns on an industrial scale, a complete transfer of the product line to an industrial scale is possible.

To guarantee the durability of the developed (hybrid) materials in outdoor applications such as a leaf spring, further investigations on water absorption should be carried out. An extensive SEM and computer tomography (CT) analysis can help identify weak points (such as voids or insufficient fibre/matrix adhesion) and further improve the materials in subsequent development.

Research is currently in progress to produce comparable yarns and fabrics from hemp. Due to the lower price of hemp from disordered lines (total fibre line) compared to flax tow, the semi-finished products’ costs could be further reduced.

## Figures and Tables

**Table 1:** Mass per unit area and composition of the fabrics (designations in brackets with Greek letters indicate internal labels which are used for the classification of the samples in the project).

Reinforcement	Mass per Unit Area in g/m^2^	Per Cent by Mass of Reinforcing Fibres in %	Per Cent by Mass of Warp Threads (PES) in %
Lincore fabric (Rε3)	275.2	84.0	16.0
Glass fabric (Rε4)	296.6	83.6	16.4
Flax fabric 006 (Aε1 and Aε3)	300.5	84.3	15.7
Flax fabric 013 (Aε4 and Aε5)	287.5	83.9	16.1

**Table 2:** Selected composites produced by different manufacturing processes from rovings, yarns and fabrics (designations in brackets with Greek letters indicate internal labels used for the classification of the samples in the project).

Pultrusion (Yarn)—Round Rods	Pultrusion (Yarn)—Rectangular Rods	Vacuum-Infusion—UD Fabric Laminates	Autoclave-Injection—UD Fabric Laminates
Lincore roving, 40% (Rγ1)	Lincore roving, 30% (Rγ1)	-	Lincore fabric (Rε3), 5 layers
Glass roving, 40% (Rγ2)	-	Glass fabric (Rε8), 6 layers	Glass fabric (Rε4), 9 layers
Flax yarn V1, 945 tex, 40% (Aγ1)	-	-	-
Flax yarn V2, 213 tex, sliver 40% (Aγ2)	Flax yarn V2, 213 tex, sliver 30% (Aγ2)	Flax fabric, yarn V2 (006, Aε3), 4 layers	Flax fabric, yarn V2 (006, Aε1), 5 layers
Flax yarn V9, 200 tex, re-stretched sliver, 40% (Aγ9)	Flax yarn V9, 200 tex, re-stretched sliver, 30% (Aγ9)	Flax fabric, yarn V9 (014, Aε5), 4 layers	Flax fabric, yarn V9 (013, Aε4), 5 layers
Flax yarn V10, 200 tex, 2x re-stretched sliver, 40% (Aγ10)	Flax yarn V10, 200 tex, 2x re-stretched sliver, 30% (Aγ10)	-	-
-	-	Flax/glass hybrid, 2 × 1 glass outer layers, 3 flax (V9) inner layers (Rε6)	Flax/glass hybrid, 2 × 2 glass outer layers, 3 flax (V9) inner layers) (Rε5)
Flax yarn, 200 tex, re-stretched sliver, 55% (Aγ9)	-	-	-

**Table 3:** Density, calculated fibre volume fraction and void content of pultruded composites (designations in brackets with Greek letters indicate internal labels which are used for the classification of the samples in the project).

Sample	Density in g/cm^3^	Fibre Volume in %	Voids in %
***Round rods***			
Lincore roving, 40% (Rγ1)	1.265 ± 0.005	45.3	2.7
Glass roving, 40% (Rγ2)	1.747 ± 0.016	46.5	1.0
Flax yarn V1, 945 tex, 40% (Aγ1)	1.228 ± 0.022	43.9	1.6
Flax yarn V2, 213 tex, sliver 40% (Aγ2)	1.270 ± 0.014	42.2	1.1
Flax yarn V9, 200 tex, re-stretched sliver, 40% (Aγ9)	1.295 ± 0.008	44.6	0.3
Flax yarn V10, 200 tex, 2x re-stretched sliver, 40% (Aγ10)	1.275 ± 0.006	43.2	1.3
Flax yarn V9, 200 tex, re-stretched sliver, 55% (Aγ9)	1.319 ± 0.005	55.5	1.1
***Rectangular rods***			
Lincore roving, 30% (Rγ1)	1.174 ± 0.004	30.4	6.9
Flax yarn V2, 213 tex, sliver 30% (Aγ2)	1.186 ± 0.017	31.5	5.4
Flax yarn V9, 200 tex, re-stretched sliver, 30% (Aγ9)	1.161 ± 0.030	32.0	7.5
Flax yarn V10, 200 tex, 2x re-stretched sliver, 30% (Aγ10)	1.152 ± 0.040	31.3	8.1

**Table 4:** Composition, density, volume fraction of polyester (PES) warp threads and calculated fibre volume fraction of composites produced by vacuum infusion and autoclave injection processes (designations in brackets with Greek letters indicate internal labels which are used for the classification of the samples in the project).

Manufacturing Process/Composite	Layers	Density in g/cm^3^	Volume Fraction PES in %	Volume Fraction Reinforcing Fibre in %
***Vacuum infusion***				
Glass fabric (Rε8)	6	1.60	11.2	30.5
Flax fabric, yarn V2 (006, Aε3)	4	1.28	6.6	33.6
Flax fabric, yarn V9 (014, Aε5)	4	1.46	7.4	36.5
Flax/glass hybrid, 2 × 1 glass outer layers, 3 flax (V9) inner layers (Rε6)	-	-	-	-
Glass	3	-	3.5	9.6
Flax	2	-	5.0	24.8
Ʃ	5	1.34	8.5	34.4
Flax/glass hybrid, 2 × 1 flax (V9) outer layers, 3 Glass inner layers (Rε7)	-	-	-	-
Glass	2	-	6.9	18.7
Flax	3	-	4.3	21.3
Ʃ	5	1.45	11.2	40.0
***Autoclave injection***				
Lincore fabric (Rε3)	5	1.34	9.6	48.0
Glass fabric (Rε4)	9	1.81	15.7	42.7 *
Flax fabric, yarn V2 (006, Aε1)	5	1.32	10.5	52.9
Flax fabric, yarn V9 (013, Aε4)	5	1.32	10.4	50.7
Flax/glass hybrid, 2 × 2 glass outer layers, 3 flax (V9) inner layers) (Rε5)	-	-	-	-
Glass	4	-	7.6	20.8
Flax	3	-	5.4	26.6
Ʃ	7	1.57	13.0	47.4

* Ashing resulted in a fibre mass fraction of 58.7%, which corresponds to a fibre volume fraction of 40.8% assuming a density of 2.6 g/cm^3^ for glass fibres.

**Table 5:** Comparison of mechanical characteristics of composite laminates (mean values ± standard deviation) (designations in brackets with Greek letters indicate internal labels which are used for the classification of the samples in the project).

Sample Mechanical Characteristics	Tensile Strength	Young’s Modulus	Flexural Strength	Flexural Modulus	Charpy impact Strength	ILSS
	in MPa	in GPa	in MPa	in GPa	in kJ/m^2^	in MPa
**Vacuum-infusion process**						
Glass fabric (Rε8), 6 layers	635.0 ± 12.3	30.1 ± 0.4	584.5 ± 43.9	18.9 ± 0.7	161.5 ± 13.1	47.7 ± 3.1
Flax fabric, yarn V2 (006, Aε3), 4 layers	165.3 ± 4.1	12.1 ± 0.5	153.9 ± 3.2	10.7 ± 0.2	25.2 ± 0.9	20.8 ± 0.6
Flax fabric, yarn V9 (014, Aε5), 4 layers	198.2 ± 6.6	16.3 ± 0.2	181.8 ± 3.6	13.4 ± 0.4	45.6 ± 3.1	28.7 ± 1.1
Flax/glass hybrid, 2 × 1 glass outer layers, 3 flax (V9) inner layers (Rε6)	315.8 ± 9.9	22.0 ± 0.3	360.9 ± 15.5	17.2 ± 0.4	124.7 ± 11.4	32.4 ± 2.6
**Autoclave-injection process**						
Lincore fabric (Rε3), 5 layers	249.4 ± 4.1	25.2 ± 0.7	232.6 ± 6.6	17.7 ± 0.5	38.8 ± 1.5	29.4 ± 0.4
Glass fabric (Rε4), 9 layers	817.3 ± 16.1	35.8 ± 1.2	452.2 ± 32.8	23.1 ± 0.2	199.0 ± 16.2	32.9 ± 0.4
Flax fabric, yarn V2 (006, Aε1), 5 layers	175.8 ± 5.7	18.8 ± 0.7	193.2 ± 3.8	12.6 ± 0.3	23.8 ± 0.9	25.4 ± 0.8
Flax fabric, yarn V9 (013, Aε4), 5 layers	215.7 ± 8.2	21.7 ± 0.8	215.1 ± 4.7	15.9 ± 0.4	42.2 ± 1.5	27.8 ± 1.1
Flax/glass hybrid, 2 × 2 glass outer layers, 3 flax (V9) inner layers) (Rε5)	401.5 ± 7.6	29.7 ± 0.8	383.9 ± 14.2	22.4 ± 0.4	182.8 ± 10.0	33.2 ± 0.5

**Table 6:** Comparison of manufactured composites’ tensile properties with other high-performance flax fibre-reinforced epoxy resins described in the literature.

Reinforcement	Fibre Volume Fraction in %	Tensile Strength in MPa	Tensile Modulus in GPa	Reference
Quasi-UD flax fabric	36	153	n.s.	[14]
Quasi-UD flax fabric (yarn V9)	37	198	16	This study
Quasi-UD Lincore fabric	40	270	28	[63]
Quasi-UD flax fabric	40	252	20	[64]
Quasi-UD flax fabric	44	163	n.s.	[14]
Quasi-UD flax fabric	45	218	23.5	[12]
Quasi-UD flax fabric	46	235	23	[6]
Quasi-UD flax fabric	47	296	27	[65]
Quasi-UD Lincore fabric	48	249	25	This study
Quasi-UD flax fabric (yarn V9)	51	216	22	This study

## Data Availability

The data presented in this study are available on request from the corresponding author.

## References

[1] Baley C., Gomina M., Breard J., Bourmaud A., Davies P. (2020). Variability of mechanical properties of flax fibres for composite reinforcement. A review. Ind. Crop. Prod..

[2] Ramesh M. (2019). Flax (*Linum usitatissimum* L.) fibre-reinforced polymer composite materials: A review on preparation, properties and prospects. Prog. Mater. Sci..

[3] Yan L., Chouw N., Jayaraman K. (2014). Flax fibre and its composites: A review. Compos. Part B Eng..

[4] Bourmaud A., Beaugrand J., Shah D.U., Placet V., Baley C. (2018). Towards the design of high-performance plant fibre composites. Prog. Mater. Sci..

[5] Scida D., Bourmaud A., Baley C. (2017). Influence of the scattering of flax fibres properties on flax/epoxy woven ply stiffness. Mater. Des..

[6] Poilane C., Cherif Z.E., Richard F., Vivet A., Doudou B., Chen J. (2014). Polymer reinforced by flax fibres as a viscoelastoplastic material. Compos. Struct..

[7] Santamala H., Livingston R., Sixta H., Hummel M., Skrifvars M., Saarela O. (2016). Advantages of regenerated cellulose fibres as compared to flax fibres in the processability and mechanical performance of thermoset composites. Compos. Part A Appl. Sci. Manuf..

[8] Shah D.U., Schubel P.J., Clifford M.J., Licence P. (2014). Mechanical property characterisation of aligned plant yarn reinforced thermoset matrix composites manufactured via vacuum infusion. Polym.-Plast. Technol. Eng..

[9] Baets J., Plastria D., Ivens J., Verpoest I. (2014). Determination of the optimal flax fibre preparation for use in unidirectional flax/epoxy composites. J. Reinf. Plast. Compos..

[10] Goutianos S., Peijs T., Nystrom B., Skrifvars M. (2006). Development of flax fibre based textile reinforcements for composite applications. Appl. Compos. Mater..

[11] Liu Q., Hughes M. (2008). The fracture behaviour and toughness of woven flax fibre-reinforced epoxy composites. Compos. Part A Appl. Sci. Manuf..

[12] Li Y., Li Q., Ma H. (2015). The voids formation mechanisms and their effects on the mechanical properties of flax fiber reinforced epoxy composites. Compos. Part A Appl. Sci. Manuf..

[13] Madsen B., Lilholt H. (2003). Physical and mechanical properties of unidirectional plant fibre composites–an evaluation of the influence of porosity. Compos. Sci. Technol..

[14] Kinloch A.J., Taylor A.C., Techapaitoon M., Teo W.S., Sprenger S. (2015). Tough, natural-fibre composites based upon epoxy matrices. J. Mater. Sci..

[15] Coroller G., Lefeuvre A., Le Duigou A., Bourmaud A., Ausias G., Gaudry T., Baley C. (2013). Effect of flax fibres individualisation on tensile failure of flax/epoxy unidirectional composite. Compos. Part A Appl. Sci. Manuf..

[16] Martin N., Davies P., Baley C. (2014). Comparison of the properties of scutched flax and flax tow for composite material reinforcement. Ind. Crop. Prod..

[17] Deimann F. (2020). Personal communication.

[18] Müssig J., Haag K., Musio S., Bjelková M., Albrecht K., Uhrlaub B., Wang S., Wieland H., Amaducci S. (2020). Biobased’ mid-performance’ composites using losses from the hackling process of long hemp–A feasibility study as part of the development of a biorefinery concept–. Ind. Crop. Prod..

[19] Zhang L., Miao M. (2010). Commingled natural fibre/polypropylene wrap spun yarns for structured thermoplastic composites. Compos. Sci. Technol..

[20] Park S.Y., Choi C.H., Choi W.J., Hwang S.S. (2019). A comparison of the properties of carbon fiber epoxy composites produced by non-autoclave with vacuum bag only prepreg and autoclave process. Appl. Compos. Mater..

[21] Sunilpete M.A., Cadambi R.M. (2020). Development of cost effective out-of-autoclave technology-vacuum infusion process with tailored fibre volume fraction. Mater. Today Proc..

[22] Prabhakaran S., Krishnaraj V., Senthil Kumar M., Zitoune R. (2014). Sound and vibration damping properties of flax fiber reinforced composites. Procedia Eng..

[23] Duc F., Bourban P.E., Plummer C.J.G., Manson J.-A.E. (2014). Damping of thermoset and thermoplastic flax fibre composites. Compos. Part A Appl. Sci. Manuf..

[24] Pil L., Bensadoun F., Pariset J., Verpoest I. (2016). Why are designers fascinated by flax and hemp fibre composites?. Compos. Part A.

[25] Lee H.P., Ng B.M.P., Rammohan A.V., Tran L.Q.N. (2017). An investigation of the sound absorption properties of flax/epoxy composites compared with glass/epoxy composites. J. Nat. Fibers.

[26] Bensadoun F., Vallons K.A.M., Lessard L.B., Verpoest I., Van Vuure A.W. (2016). Fatigue behaviour assessment of flax-epoxy composites. Compos. Part A Appl. Sci. Manuf..

[27] Gassan J. (2002). A study of fibre and interface parameters affecting the fatigue behaviour of natural fibre composites. Comosites Part A Appl. Sci. Manuf..

[28] Asgarinia S., Viriyasuthee C., Phillips S., Dube M., Baets J., Van Vuure A., Verpoest I., Lessard L. (2015). Tension-tension fatigue behaviour of woven flax/epoxy composites. J. Reinf. Plast. Compos..

[29] Seghini M.C., Touchard F., Sarasini F., Chocinski-Arnault L., Ricciardi M.R., Antonucci V., Tirillo J. (2020). Fatigue behaviour of flax-basalt/epoxy hybrid composites in comparison with non-hybrid composites. Int. J. Fatigue.

[30] Dhakal H.N., Zhang Z.Y., Guthrie R., MacMullen J., Bennett N. (2013). Development of flax/carbon fibre hybrid composites for enhanced properties. Carbohydr. Polym..

[31] Fiore V., Valenza A., Di Bella G. (2012). Mechanical behavior of carbon/flax hybrid composites for structural applications. J. Compos. Mater..

[32] Ravandi M., Kureemun U., Banu M., Teo W.S., Tong L., Tay T.E., Lee H.P. (2019). Effect of interlayer carbon fiber dispersion on the low-velocity impact performance of woven flax-carbon hybrid composites. J. Compos. Mater..

[33] Sarasini F., Tirillo J., D’Altilia S., Valente T., Santulli C., Touchard F., Chocinski-Arnault L., Mellier D., Lampani L., Gaudenzi P. (2016). Damage tolerance of carbon/flax hybrid composites subjected to low velocity impact. Compos. Part B Eng..

[34] Wang A., Wang X., Xian G. (2020). Mechanical, low-velocity impact, and hydrothermal aging properties of flax/carbon hybrid composite plates. Polym. Test..

[35] Papa I., Ricciardi M.R., Antonucci V., Pagliarulo V., Lopresto V. (2018). Impact behaviour of hybrid basalt/flax twill laminates. Compos. Part B Eng..

[36] Petrucci R., Santulli C., Puglia D., Sarasini F., Torre L., Kenny J.M. (2013). Mechanical characterisation of hybrid composite laminates based on basalt fibres in combination with flax, hemp and glass fibres manufactured by vacuum infusion. Mater. Des..

[37] Savran M., Aydin L. (2018). Stochastic optimisation of graphite-flax/epoxy hybrid laminated composite for maximum fundamental frequency and minimum cost. Eng. Struct..

[38] Sarwar A., Mahboob Z., Zdero R., Bougherara H. (2020). Mechanical characterisation of a new kevlar/flax/epoxy hybrid composite in a sandwich structure. Polym. Test..

[39] Barouni A.K., Dhakal H.M. (2019). Damage investigation and assessment due to low-velocity impact on flax/glass hybrid composite plates. Compos. Struct..

[40] Calabrese L., Fiore V., Scalici T., Valenza A. (2019). Experimental assessment of the improved properties during aging of flax/glass hybrid composite laminates for marine applications. J. Appl. Polym. Sci..

[41] Fiore V., Calabrese L., Scalici T., Bruzzaniti P., Valenza A. (2018). Bearing strength and failure behavior of pinned hybrid glass-flax composite laminates. Polym. Test..

[42] Jusoh M.S.M., Santulli C., Yahya M.Y., Hussein N.S., Ahmad H. (2017). Effect of stacking sequence on the tensile and flexural properties of glass fibre epoxy composites hybridised with basalt, flax or jute fibres. Mater. Sci. Eng. Adv. Res..

[43] Meenakshi C.M., Krishnamoorthy A. (2019). Study on the effect of surface modification on the mechanical and thermal behaviour of flax, sisal and glass fiber-reinforced epoxy hybrid composites. J. Renew. Mater..

[44] Morye S.S., Wool R.P. (2005). Mechanical properties of glass/flax hybrid composites based on a novel modified soybean oil matrix material. Polym. Compos..

[45] Saidane E.H., Scida D., Assarar M., Sabhi H., Ayad R. (2016). Hybridisation effect on diffusion kinetic and tensile mechanical behaviour of epoxy based flax-glass composites. Compos. Part A Appl. Sci. Manuf..

[46] Saidane E.H., Scida D., Assarar M., Ayad R. (2017). Damage mechanisms assessment of hybrid flax-glass fibre composites using acoustic emission. Compos. Struct..

[47] Santulli C., Janssen M., Jeronimidis G. (2005). Partial replacement of e-glass fibers with flax fibers in composites and effect on falling weight impact performance. J. Mater. Sci..

[48] Selver E., Ucar N., Gulmez T. (2018). Effect of stacking sequence on tensile, flexural and thermomechanical properties of hybrid flax/glass and jute/glass thermoset composites. J. Ind. Text..

[49] Sathish S., Kumaresan K., Prabhu L., Gokulkumar S. (2018). Experimental investigation of mechanicaland ftir analysis of flax fiber/epoxy compositesincorporating sic, Al_2_O_3_ and graphite. Rom. J. Mater..

[50] Zhang Y., Li Y., Ma H., Yu T. (2013). Tensile and interfacial properties of unidirectional flax/glass fiber reinforced hybrid composites. Compos. Sci. Technol..

[51] Deutsches Institut für Normung (2005). DIN EN ISO 139:2005–Textiles-Standard Atmospheres for Conditioning and Testing.

[52] Graupner N., Sarasini F., Müssig J. (2020). Ductile viscose fibres and stiff basalt fibres for composite applications-an overview and the potential of hybridisation. Compos. Part B Eng..

[53] Deutsches Institut für Normung (2005). DIN EN ISO 291:2005–Plastics–Standard Atmospheres for Conditioning and Testing.

[54] Deutsches Institut für Normung (2003). DIN EN ISO 14125:2003–Fibre-Reinforced Plastic Composites–Determination of Flexural Properties.

[55] International Organization for Standardization (2003). ISO 3597-1:2003: Textile-Glass-Reinforced Plastics–Determination of Mechanical Properties on Rods Made of Roving-Reinforced Resin–Part 2: Determination of Flexural Strength.

[56] Deutsches Institut für Normung (1998). DIN EN 14130:1998–Fibre-Reinforced Plastic Composites–Determination of Apparent Interlaminar Shear Strength by Short-Beam Method.

[57] Deutsches Institut für Normung (1996). DIN EN ISO 527-2:1996–Plastics–Determination of Tensile Properties–Part 2: Test Conditions for Moulding and Extrusion Plastics.

[58] Truong M., Zhong W., Boyko S., Alcock M. (2009). A comparative study on natural fibre density measurement. J. Text. Inst..

[59] Van de Weyenberg I., Ivens J., De Coster A., Kino B., Baetens E., Verpoest I. (2003). Influence of processing and chemical treatment of flax fibres on their composites. Compos. Sci. Technol..

[60] Heijenrath R., Peijs T. (1996). Natural-fibre-mat-reinforced thermoplastic composites based on flax fibres and polypropylene. Adv. Compos. Lett..

[61] Riedel U., Gassan J., Karus M., Müssig J., Prömper E., Schönberger D., Sperber V. (2005). Technical and economical framework for parts based on natural fibre composites. Proceedings of the 8th Internationale AVK-TV Tagung für verstärkte Kunststoffe und duroplastische Formmassen.

[62] Cerbu C., Botis M. (2017). Numerical modeling of the flax/glass/epoxy hybrid composite materials in bending. Procedia Eng..

[63] Groupe Depestele (2020). Dry Flax Fabrics Range. https://www.groupedepestele.com/pro_ecomateriaux_licoreffang.html.

[64] Duc F., Bourban P.E., Manson J.-A.E. (2014). The role of twist and crimp on the vibration behaviour of flax fibre composites. Compos. Sci. Technol..

[65] Kersani M., Lomov S.V., Van Vuure A.W., Bouabdallah A., Verpoest I. (2015). Damage in flax/epoxy quasi-unidirectional woven laminates under quasi-static tension. J. Compos. Mater..

[66] Compston P., Jar P.-Y.B. (1999). The influence of fibre volume fraction on the mode I interlaminar fracture toughness of a glass-fibre/vinyl ester composite. Appl. Compos. Mater..

[67] Budan A.D., Basavarajappa S., Prasanna Kumar M., Joshi A.G. (1999). Influence of fibre volume reinforcement in drilling GFRP laminates. J. Eng. Sci. Technol..

[68] Marrot L., Bourmaud A., Bono P., Baley C. (2014). Multi-scale study of the adhesion between flax fibers and biobased thermoset matrices. Mater. Des..

[69] Esnaola A., Tena I., Aurrekoetxea J., Gallego I., Ulacia I. (2016). Effect of fibre volume fraction on energy absorption capabilities of e-glass/polyester automotive crash structures. Compos. Part B Eng..

[70] Ashir M., Nocke A., Bulavinov A., Pinchuk R., Cherif C. (2019). Influence of defined amount of voids on the mechanical properties of carbon fiber-reinforced plastics. Polym. Compos..

[71] Dong C. (2016). Effects of process-induced voids on the properties of fibre reinforced composites. J. Mater. Sci. Technol..

[72] Zhou Y., Huang Z.-M., Liu L. (2019). Prediction of interfacial debonding in fiber-reinforced composite laminates. Polym. Compos..

[73] Graupner N., Labonte D., Müssig J. (2017). Rhubarb petioles inspire biodegradable cellulose fibre-reinforced PLA composites with increased impact strength. Compos. Part A Appl. Sci. Manuf..

[74] Graupner N., Labonte D., Humburg H., Buzkan T., Dörgens A., Kelterer W., Müssig J. (2017). Functional gradients in the pericarp of the green coconut inspire asymmetric fibre-composites with improved impact strength, and preserved flexural and tensile properties. Bioinspiration Biomim..

[75] Thomason J.L., Vlug M.A. (1997). Influence of fibre length and concentration on the properties of glass fibre-reinforced polypropylene. 4. Impact properties. Compos. Part A Appl. Sci. Manuf..

[76] Ashby M.F., Gibson L., Wegst U., Olive R. (1995). The mechanical properties of natural materials. I. material property charts. Proc. R. Soc. Lond. A.

[77] Ashby M.F., Jones D.R.H. (2005). Werkstoffe 2: Metalle, Keramik und Gläser, Kunststoffe und Verbundwerkstoffe.

